# Improving outcomes for patients with lymphoma: design and development of the Australian and New Zealand Lymphoma and Related Diseases Registry

**DOI:** 10.1186/s12874-022-01728-0

**Published:** 2022-10-10

**Authors:** Lymphoma and Related Diseases Registry Investigators, Mary Ann Anderson, Leanne Berkahn, Chan Cheah, Michael Dickinson, Maher K. Gandhi, Pratyush Giri, Eliza A. Hawkes, Anna Johnston, Colm Keane, Zoe K. McQuilten, Stephen P. Mulligan, Stephen Opat, Dipti Talaulikar, Judith Trotman, Janne Williams, Erica M. Wood, Tasman Armytage, Allison Barraclough, Duncan Carradice, Geoffrey Chong, Tara Cochrane, Nada Hamad, Matthew Ku, Denise Lee, Susan Morgan, Howard Mutsando, Manjunath Narayana, H. Miles Prince, Sumita Ratnasingam, Joel Wight, Xavier Badoux, Gavin Cull, Bryone Kuss, Paula Marlton, Constantine Tam, Joshua Casan, Tania Cushion, Aditya Tedjaseputra, Simone Birch, Christina Brown, David Ellis, Yasmin Harvey, Sam Hitchins, Sanjiv Jain, Peter Jessup, Surender Juneja, Daniel Kearney, Beena Kumar, Stephen Lade, Kenneth Lee, Connull Leslie, Eileen Long, Adrienne Morey, Lakshmi Nath, Debra Norris, Andrew Parker, Jeremy Parry, Fiona Pin-Yen Chen, Eliza Chung, Jessica Morison, Luke Rowsell, Gayathri St George, Christianto Thu, Neil Waters, Cameron Wellard, Michelle Zheng

**Affiliations:** Lymphoma and Related Diseases Registry, School of Public Health and Preventive Medicine, Monash University, 553 St Kilda Road, Melbourne, Victoria 3004 Australia

**Keywords:** Lymphoma, Chronic lymphocytic leukaemia, Clinical quality registry, Epidemiology

## Abstract

**Background:**

Lymphoma is a malignancy of lymphocytes and lymphoid tissues comprising a heterogeneous group of diseases, with up to 80 entities now described. Lymphoma is the 6^th^ most common cancer in Australia, affecting patients of all ages, with rising incidence rates. With the proliferation of efficacious novel agents, therapeutic strategies are increasingly diverse and survival is improving. There is a clear need for contemporary robust and detailed data on diagnostic, investigational and management strategies for this disease in Australia, New Zealand and worldwide, to inform and benchmark local and international standards of care. Clinical quality registries can provide these data, and support development of strategies to address variations in management, including serving as platforms for clinical trials and other research activities. The Lymphoma and Related Diseases Registry (LaRDR) was developed to capture details of patient demographics, disease characteristics, and management throughout their disease course and therapy and to develop outcome benchmarks nationally and internationally for lymphoma. This report describes the aims, development and implementation of the LaRDR, as well as challenges addressed in the process.

**Methods:**

The LaRDR was established in 2016 as a multicentre, collaborative project at sites across Australia with a secure online database which collects prospective data on patients with a new diagnosis of lymphoma or chronic lymphocytic leukaemia (CLL). LaRDR development required multidisciplinary participation including specialist haematology, information technology, and biostatistical support, as well as secure funding. Here we describe the database development, data entry, ethics approval process, registry governance and support for participating sites and the coordinating centre.

**Results:**

To date more than 5,300 patients have been enrolled from 28 sites in Australia and New Zealand. Multiple challenges arose during the development, which we describe, along with approaches used to overcome them. Several confirmed international collaborations are now in place, and the registry is providing valuable data for clinicians, researchers, industry and government, including through presentations of results at major national and international conferences.

**Conclusion:**

Challenges in establishing the LaRDR have been successfully overcome and the registry is now a valuable resource for lymphoma clinicians, researchers, health economists and others in Australia, New Zealand and globally.

**Supplementary Information:**

The online version contains supplementary material available at 10.1186/s12874-022-01728-0.

## Introduction

The Lymphoma and Related Diseases Registry (LaRDR) was established in 2016 with the aim to improve the quality of care and clinical outcomes for people with lymphoma and chronic lymphocytic leukaemia (CLL), through systematic collection, analysis and reporting of real-world data to understand lymphoma epidemiology, current management and outcomes in Australia and New Zealand. Here we describe the rationale, development process and initial experience from the registry.

### The value of clinical registries

Clinical quality registries (CQR) are now well established internationally, and endorsed as integral to continuous improvement in healthcare through supporting delivery of and monitoring evidence-based practice [1]. Key features of CQRs and their value, including for blood cancers, are reviewed in detail elsewhere [2–4]. By collecting a standard minimum dataset, which increasingly includes patient-reported outcomes, registries are also very valuable for uncommon diseases or interventions where clinical trials are challenging, and even large referral centres may see few patients. In this context registries can provide a mechanism to identify variation in practice. They also and serve as efficient platforms to conduct observational studies and interventional trials to establish optimal management and conduct health economics analyses using ‘real world’ data [2–4]. Linkage with other datasets, such as cancer and death registries, can also be readily undertaken.

### A clinical quality registry for lymphoma

Lymphomas are cancers of lymphocytes and lymphoid tissues – the lymph nodes and related organs, such as the spleen. These cancers are classified according to their cell of origin and increasingly by molecular diagnostics, with more than 80 entities now recognised [5]. Lymphoma is the sixth most common cancer diagnosis in Australia with more than 6000 new diagnoses annually, and the incidence is rising [6]. CLL is the single most common lymphoid cancer in adults with over 2000 new cases reported annually in Australia. Its long natural history of asymptomatic disease, with many never requiring treatment, and unique features compared with other lymphoid cancers highlighted the desirability of a CLL-specific module (see below).

Lymphoid malignancies affect people of all ages, and impose a significant burden for patients and the health system, with high rates of hospitalisations for treatment delivery and for management of complications, such as infection [6]. Therapies are often complex, and must be tailored to the specific type of lymphoid cancer with many patients undergoing multiple lines of therapy during the course of their disease; management may include a combination of chemotherapy, immunotherapy, small molecule drugs, radiation, cellular therapies such as autologous or allogeneic haematopoietic stem cell transplant or chimeric antigen-receptor T-cell therapeutics, and occasionally surgery, along with supportive care measures such as immunoglobulin replacement therapy and transfusions. Survival is improving likely due to improvements in diagnosis, better supportive care, and the availability of new targeted therapies, but many of these are costly, and also carry specific adverse effect profiles.

Few Australian data are available on lymphoma treatments and outcomes outside the setting of clinical trials, and fewer than 5% of adult cancer patients are enrolled on clinical trials [7]. State cancer diagnosis registries can provide important but limited data on diagnoses and deaths, but no information on patient factors such as comorbidities, treatment or outcomes other than death, including quality of life. CQRs can help address many of the substantial evidence gaps that need to be addressed to better inform policy and improve practice and outcomes.

With increasing complexity of diagnosis and management, a need was identified for contemporary national Australian epidemiological, therapy, clinical outcome and health economic data for lymphoma and CLL to complement clinical trials, and a lymphoma CQR was proposed.

## Methods

### Governance

A steering committee oversees LaRDR activities and provides research and project guidance according to documented Terms of Reference. Members include clinicians from across Australia and New Zealand based on their expertise in lymphoma and CLL diagnosis and management, and to provide broad geographic representation, along with epidemiologists, registry experts and patient representation. The steering committee meets three times per year, and as required, with other business being conducted as necessary between meetings. Data access, publication and other relevant policies are in place.

LaRDR is managed by the School of Public Health and Preventive Medicine at Monash University, a large academic organisation with expertise in clinical quality registries, in partnership with participating hospitals and clinicians. Site investigators oversee activities at participating hospitals. A multidisciplinary project team (project managers, data managers, registry experts, lymphoma clinician) coordinates day-to-day activities, and provides support to the steering committee and site staff and investigators.

### Funding

The registry is supported by multiple industry partners, on a sponsorship and/or project basis. These partners can request targeted analyses and reports based on their interests, but do not direct the overall research activities of the registry. Industry funding is acknowledged as a potential conflict of interest in presentations and publications. A modest per patient payment to sites supports data entry activities.

### Ethics approval and consent to participate

The LaRDR has human research ethics committee (HREC) approval from Monash Health (HREC 16/MonH/74) and all participating hospital sites, now (since 2016) under a national mutual acceptance (NMA) ethics scheme which allows publicly funded health services across all jurisdictions to accept an ethical review from an external accredited HREC. NMA arrangements were in place for clinical trials but not for registries at the time of commencing work on LaRDR, necessitating time-consuming HREC applications to all initial sites individually. Local governance approvals are still required to ensure sites can support the project activity. In 2022, the registry expanded to New Zealand following approval by the New Zealand Health and Disability Ethics Committees (reference: 2022 FULL 12203, 29 March 2022).

LaRDR utilises an “opt out” consent model, an approach approved by the Australian National Health and Medical Research Council and New Zealand National Ethics Advisory Committee if the public interest in a research study sufficiently outweighs the potential impingement on individual privacy. This model enables maximum participation and thereby reduces bias; it is widely used for registry activities in Australia. Clinicians at participating sites are responsible for identifying potential participants, explaining the study to them, inviting them to participate, and providing them the approved LaRDR information brochure, which describes the registry aims, data being collected and LaRDR contact details. This process is documented in the patient’s file in the registry. No written consent is required. Patients may opt out at any time from initial invitation or in the future, at which point any of that person’s data will be deleted centrally. The consent also provides for centralised review of laboratory results and histology slides.

Registry analyses by approved investigators using existing LaRDR data can be conducted without additional HREC approval. Sub-studies requesting additional data typically require additional approval.

The project is registered on the Australian and New Zealand Clinical Trials Registry (ACTRN12617000050358).

### Patient selection

Patients 18 years or older, with diagnoses of any type of non-Hodgkin lymphoma, Hodgkin lymphoma, CLL or related diseases in accordance with the WHO classification [5], are eligible to participate. The registry collects prospective data on incident cases – a case being defined as having received a diagnosis subsequent to or within 6 months prior to the participating site securing HREC approval to participate in LaRDR, in order to minimise selection bias and the burden of retrospective data collection and to maximise data completeness. An exception is made for CLL, which is frequently slowly progressive, and a significant proportion of patients may never require CLL-directed therapy. Therefore, retrospective data on CLL patients diagnosed up to 10 years previously can be included, provided complete data are available. For deceased patients where the cause of death is listed as lymphoma or CLL a waiver of consent is in place to obtain data.

### Establishing a minimum dataset

Data items included in the minimum dataset are listed in Table 1. Datasets and case report forms for lymphoma and CLL were designed by the steering committee and project team, and refined iteratively. A CLL-specific case report form was developed due to the particular staging, disease trajectory and therapeutic paradigm applicable to this disease subtype. Data dictionaries are available for reference.

**Table 1:** Key data items and time-points

Key data entry time points	Data items collected
Baseline: Demographics and disease characteristics at diagnosis	Date of birth, sex, genetic ethnic heritage, pregnancy status Height and weight Medical history, including current comorbidities, previous malignancies, ECOG performance status Family history of haematologic malignancy WHO classification disease stage Molecular and cytogenetic abnormalities – Those judged to be of most prognostic value for a given diagnosis Full blood count Other laboratory fields relevant to prognostic indices Quality of life (EQ-5D-5L)^a^ Samples tissue banked, if applicable
Therapy (Repeatable event to collect each line)	Planned therapy, including chemo-/immuno-/radiotherapy, haematopoietic stem cell transplantation (autologous/allogeneic), supportive care, participation in clinical trial (if applicable) Delivered therapy, including commencement date and any variations to planned therapy Response: Interim response and initial response
Reviews (6 and 12 months and annually thereafter or as required)	Vital status, date and cause of death if applicable Relapse/progression, date of progression if appropriate Loss to follow-up, date of last contact

*ECOG* Eastern Cooperative Oncology Group, *EQ-5D-5L* EuroQol 5D 5-level QoL instrument, *WHO* World Health Organization, ^a^Ethics approval in place but currently not collected

The minimum data set includes information on demographics, comorbidities, diagnosis, planned therapy (if any) and supportive care, which are collected at baseline, with relevant updates plus disease response and survival entered at 6 and 12 months, and annually thereafter. Quality of life and biobanking data options were included to accommodate future projects. Data items are added (or deleted if not needed or feasible to collect) with approval of the Steering Committee and LaRDR data manager.

### Data management, quality control, and analysis

LaRDR uses a REDCap database hosted and managed by Helix at Monash University. REDCap (Research Electronic Data Capture) is a secure, web-based software platform designed to support data capture for research studies, providing 1) an intuitive interface for validated data capture; 2) audit trails for tracking data manipulation and export procedures; 3) automated export procedures for seamless data downloads to common statistical packages; and 4) procedures for data integration and interoperability with external sources [8, 9].

The database has a user-friendly interface and requires only basic training for site staff. To minimise data entry error, and aid analysis, most fields were designed to be dropdown, check boxes or radio buttons with minimum free text requirements, since data collection is typically performed by non-medically trained staff who rely on hospital electronic and paper patient medical records and may not be familiar with specific disease- or treatment-related details. Clarification on specific items can be sought from lymphoma and CLL experts on the project team, and/or site investigators.

LaRDR project staff conduct quality control activities, review data queries, and provide feedback and reports to site staff, investigators and the steering committee. A data validation committee reviews inconsistencies to refine definitions, data fields and user instructions, and conducts audits to review data completeness and accuracy. Sites may access their own data at any time and can manage local reports to facilitate local audits and data completion.

Students, medical specialists in training and others undertake research using registry data. All research projects must be approved by the steering committee who provide oversight to all approved projects, ensuring no overlap between projects and to help ensure timely completion. In accordance with the LaRDR data access policy, projects that require patient-level data access this via Monash University’s secure environment for sharing research data (SeRP), a secure platform that allows researchers to analyse de-identified, patient-level data. Results from these analyses must first be approved by the data custodian before they can be exported, with only aggregate data approved for export. LaRDR staff are available to provide statistical support as well clinical insight to all projects. Data are published in an aggregate form.

Data linkage with state and national cancer registry data are planned to ensure that all eligible patients at participating sites are captured, and that missing or discrepant cases are followed up with sites. Annual linkage with the National Death Index in each country is planned to validate survival data.

### Working groups

1. Pathology review working group: There are over 80 recognised subtypes of lymphoma with distinct biology and clinical behaviour, and sub-classification of lymphoma is a complex process based on a combination of clinical, morphologic, cytometric , cytogenetic and molecular features. Accurate diagnosis and documentation is essential for interpretation of data reported to the registry, and the Pathology Review working group has an important role ensuring that cases are appropriately categorised. This national committee consisting of anatomical pathologists and haematologists advises on data collection and interpretation, and can provide centralised pathology review for clinical studies and trials. If results are uncertain or discrepant, directors of pathology departments at participating sites can be contacted to recommend local review.
2. CLL working group: CLL diagnosis, prognostication and management has now diverged significantly from non-Hodgkin lymphoma. Not all patients require treatment, but for those who do, the optimal use of newer therapies, including combinations and sequencing of agents, is yet to be defined. Furthermore emerging evidence supporting a key prognostic role for genetic and measurable residual disease testing in this condition requires an evidence base to support its optimal clinical application. A dedicated working group comprising 11 CLL experts designed the CLL-specific dataset and data fields, which was integrated into the existing LaRDR database and tested before being made accessible to other registry users.
3. Data validation committee: The management of lymphoma is rapidly evolving, with new treatment protocols and diagnostic tests continually emerging. The role of the data validation committee, made up of lymphoma and registry experts, is to ensure the registry keeps pace with this evolution by reviewing the data fields that are collected and updating them as appropriate.

### Communications and reporting

Hospital data reports are provided annually to individual sites, with site-specific, aggregate de-identified patient data presented and compared with overall national data. A breakdown of major diagnostic groups and their characteristics, treatment and survival data, and information on data completeness, is included. This allows benchmarking with other health services nationally and participating hospitals can identify site-specific issues for clinical audit and further investigation. Sites with low patient recruitment receive generic reports until sufficient data have accrued (see example: Additional file 1).

Summary LaRDR annual reports are published on the LaRDR website (lardr.org). Annual open meetings, usually conducted in conjunction with the national haematology scientific congresses, or virtually in 2020-21, provide opportunities for clinicians, site staff, industry partners and students to learn more about the registry. Scientific results are presented at local and international conferences and published in the peer-reviewed literature [10–13]. Commissioned reports are also provided to industry partners and may be requested by others (for example, government agencies).

## Results

### Pilot phase and activities

**Fig. 1:**
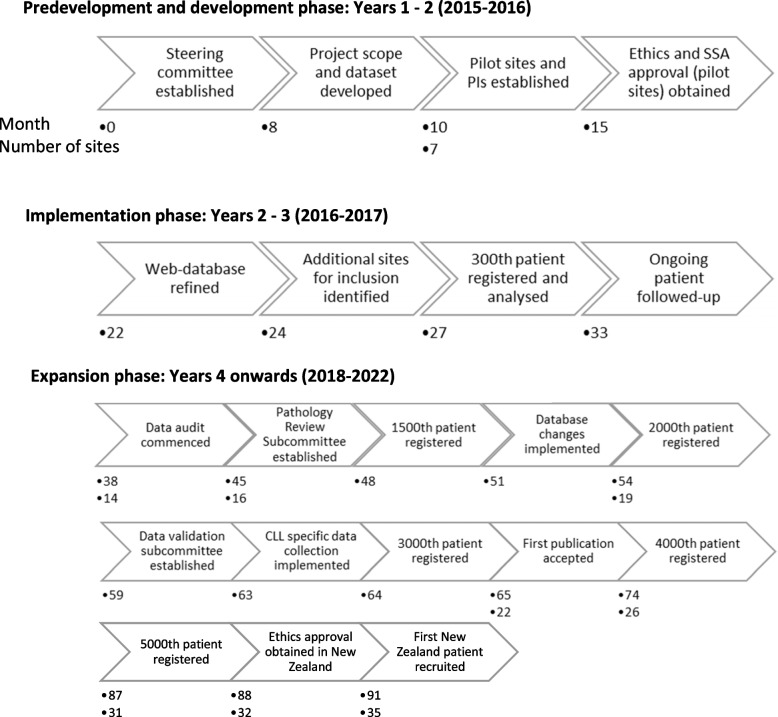
Timeline of registry development, implantation and expansion phase

The registry commenced with a pilot in 2016 with 6 large metropolitan hospitals in Australia with lymphoma expertise and resources and who had expressed interest in participating. These sites and their teams were crucial in planning, testing and providing feedback on all aspects of the registry, including governance and operations, and refining the minimum dataset, data entry processes and the database. Data completeness reports were generated and fields with low completion rates reported back to data managers and compared with detailed information from site staff on data that were onerous to find in medical records or where instructions were unclear. Results were discussed by the steering committee and a number of important changes made to the database and processes based on this feedback. An indicative timeline of registry establishment and progress is given in Fig. 1.

### Current status

**Fig. 2:**
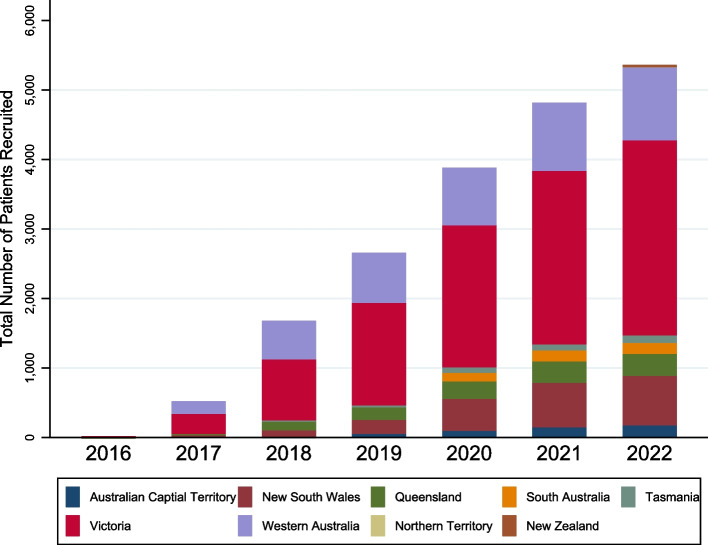
LaRDR recruitment by Australian states and territories and New Zealand from June 2016 to August 2022

Across six Australian states and two territories, and one New Zealand site, more than 5300 patients are currently enrolled from 28 sites, with 33 hospitals open to recruitment and a further 2 sites awaiting governance approval; others have also expressed interest in joining. Whilst still dominated by large tertiary metropolitan centres, site profiles are diversifying, with the addition of five regional hospitals and one private hospital since the pilot phase. Recruitment to date is shown in Fig.  2 and the frequency of cases according to major disease group in Table 2. National Australian coverage is currently estimated at 20% of lymphoma cases diagnosed annually, and continuing to expand. All sites in Australia and New Zealand are now welcome to join the registry.

**Table 2:** Baseline diagnostics and demographic characteristics

Age (years), median (IQR)	64.3 (52.1-73.5)
Follow-up time months^a^, median (IQR)	18.4 (10.5-26.1)
Gender	
Male	3165/5307 (59.6%)
Female	2142/5307 (40.4%)
ECOG performance status	
0	2374/3925 (60.5%)
1	1110/3925 (28.3%)
2	279/3925 (7.1%)
3	119/3925 (3.0%)
4	43/3925 (1.1%)
Diagnosis	
Diffuse large B cell lymphoma (DLBCL)	1717/5314 (32.3%)
Follicular lymphoma	816/5314 (15.4%)
Other B cell non-Hodgkin lymphoma	909/5314 (17.1%)
Hodgkin Lymphoma	751/5314 (14.1%)
Chronic lymphocytic leukaemia (CLL)	560/5314 (10.5%)
T-cell non-Hodgkin lymphoma	283/5314 (5.3%)
Mantle cell lymphoma	249/5314 (4.7%)
Post-transplant lymphoproliferative disease	29/5314 (0.5%)

^a^For prospectively identified patients only

Some key diagnostic and demographic data are presented in Table 2. Median follow-up time for prospectively enrolled patients on the registry is 18 months, with 35% of patients having more than two-years follow-up. As the data mature, follow-up times will increase which is important given the long disease course and excellent prognosis of some lymphoma subtypes and CLL. The first data linkage with the Victorian Cancer registry is currently underway, and we plan to begin annual linkages with the National Death Index to validate mortality data and improve estimates of overall survival, a key endpoint in many analyses.

The registry has already generated interest among the international lymphoma clinical and patient communities, government, and industry partners. To date, 20 research projects have been completed or are underway using registry data and infrastructure, with three international collaborations now formalised, delivery of 26 national and international conference presentations, and provision of 20 data reports to industry, investigators and government, along with publication of a manuscript (see: lardr.org/research/#Researchpublications and [10–13]).

## Discussion

LaRDR is now established and delivering new national data on lymphoma and CLL epidemiology, management and outcomes. By describing and sharing our experiences, we hope that this will assist others planning similar activities, as we ourselves have built on the experience of the project team and investigators, including previously setting up the Australian and New Zealand Myeloma and Related Diseases Registry and other registries [4].

Some of the challenges with establishing LaRDR are applicable to all registries (and many other types of major research infrastructure projects). These are summarised in Table 3. Other aspects of the early LaRDR experience also hold lessons for establishing registries for other complex conditions. These include confirming the initial diagnosis: lymphoma diagnosis and staging is complex, requiring a set of coordinated clinical, imaging and pathology investigations, some of which are specific to particular disease subtypes. Even expert pathologists can disagree on diagnostic assignments in lymphoma, and this is even more challenging in a national registry setting since it is not practical to require review of primary diagnostic material for all cases from every site – and there would be implications for changing a diagnosis after subsequent review where the patient has already received therapy. Managing the diversity of diagnoses (and accounting for changing diagnoses where low-grade diseases transform to a more aggressive form), and periodic updates to the WHO classification, with implications for existing and new entries in the database, adds further complexity. Furthermore, the registry was established to collect data prospectively, intending to enhance data completeness and reduce bias. However, CLL and some types of lymphoma are typically very indolent in their disease course, with little change in status over years or even decades. Patients with stable CLL managed with ‘watch and wait’ approach are not captured in clinical trials (as by definition they do not require treatment), but nevertheless have disease complications such as immune failure, and autoimmune disease. These patients can also be markedly under-represented in registry data. The long-term follow-up is also important to capture complications such as second malignancy that tend to occur more commonly over time [14]. Furthermore, with the dramatic improvements over the last decade with immunochemotherapy and then novel therapies, prolonged survival is much more commonly seen than previously and these long-term complications related to this improved survival will be important to document as the ‘new natural history’ of CLL. To address these important questions, the CLL group allowed retrospective data entry for CLL where sites were confident of access to complete data.

**Table 3:** Common challenges to registry development encountered and addressed

Challenge	Resolution	Examples
**Generating and sustaining engagement, demonstrating value**	Highlighting advantages for: **• Patients:** Knowing their data will help create a national picture of the condition, and that their hospital is participating in benchmarking for best practice	**•** Patient representation on the Steering Committee **•** Newsletters, website information and presentations for patient/community groups
	**• Sites and clinicians:** Access to own and other data for comparison and benchmarking, access to a peer network, opportunities for projects for young investigators, access to data on rare conditions where trials are difficult	**•** Direct access to own data **•** Hospital data reports **•** Participation in registry committees **•** Research project opportunities, supported by LaRDR team
	**• Industry, policy-makers and others:** Access to real world data including on treatment sequencing, uptake of new, high-cost or high-risk therapies, reasons for discontinuation, data to support regulatory applications and health service planning	**•** Presentations at scientific meetings and to government **•** Annual open investigator meetings **•** Peer-reviewed publications **•** Commissioned reports
**Ethics and governance**	**• ‘Opt out’ consent model:** works well for engaging patients and obtaining clinical data but unfamiliar to some (including some HREC & governance committees) **• Historically fragmented HREC system:** time-consuming to apply individually to local HREC and governance committees	**•** Engagement with site HREC and governance committees **•** Seek written consent in future to obtain biological samples **•** Transition to national mutual acceptance ethics arrangements
**Sustainability**	**• Pilot phase and managed roll-out:** Essential to recognise and address operational problems early, but few results generated during this stage **• Site resources**: generally very limited, alleviated somewhat by per-patient payments provided initially **• Secure ongoing funding:** always challenging until data mature and can generate analyses and peer-reviewed publications	**•** Respond to feedback, and keep stakeholders informed of progress and plans **•** Per-patient payments: even modest support is valuable and permits managers to allocate staff time for data entry **•** Communicate potential of registry data and how industry, clinicians, researchers, government agencies can access
**Data and access**	**• Data entry burden:** Find a balance between collecting all possible data, determining what is feasible to collect, and what will actually be used –determining an initial minimum dataset for the registry, which is subject to ongoing review and potential for additions (e.g. patient-reported outcomes)	**•** Include stakeholders in planning to consider feasibility: registry scope and the content of the dataset, noting that data collectors will likely not be experts in this field **•** Provide training for site staff and access to ongoing support and resources, and data definitions **•** Data validation committee and periodic audits of data completeness and utilisation **•** Per-patient payments for data
	• **Maximising data access while maintaining data security**	Use of institutional secure e-research platform (SeRP) permits authorised users to access and analyse data within a data ‘safe haven’, under control and supervision of registry data custodian
**Research activities**	**• Promoting research**, especially early and before many patient outcomes available	Clear guidelines on participation Promote project and authorship opportunities Inclusivity Supporting younger researchers Annual research meetings

### Future directions

LaRDR is now an established CQR. It is well placed to continue its expansion with increased national coverage in both Australia and New Zealand, and to support future research, including by publishing results of analyses and providing epidemiological data (such as numbers and geographic location of patients with data on diagnoses and disease stage) which will inform planning of clinical trials. The registry can also serve as a platform for conducting clinical trials [15] and observational studies, and enable efficient, long-term follow up after these studies have been completed. In addition to information contained within the registry itself, LaRDR data can be used for epidemiological modelling and linkage activities to inform policy development and health service planning, especially for new and high-cost therapies and to ensure improved access to and delivery of care for all patients.

## Supplementary Information

**Additional file 1.**

## Data Availability

Access to data that support the findings of this study are available from the Lymphoma and Related Diseases Registry with permission from the Steering Committee and in accordance with the LaRDR Data Access Policy. More information is available at lardr.org or via email to: SPHPM-Lymphoma@monash.edu
