# Supplementary material for: Improving outcomes for patients with lymphoma: design and development of the Australian and New Zealand Lymphoma and Related Diseases Registry
Source: BMC Med Res Methodol. 2022 Oct 10;22:266. doi: 10.1186/s12874-022-01728-0 (PMC9549605; doi:10.1186/s12874-022-01728-0)
Supplement: Supplementary file 1 — Additional file 1. [file 12874_2022_1728_MOESM1_ESM.pdf]

Lymphoma

and

Related Diseases Registry

LaRDR Data Report

For period ending 05 November 2021

Prepared November 2021

Lymphoma and Related Diseases Registry team

Monash University

Dr Cameron Wellard, Ms Gaya St George, Ms Eliza Chung, Dr Fiona Chen, Mr Neil Waters, A/Prof Eliza Hawkes, A/Prof Zoe McQuilten, Prof Erica Wood, Prof Stephen Opat

Contents

Introduction 3
Site status at time of report 3
LaRDR sites across Australia and New Zealand 4
Case accrual 5
Hodgkin lymphoma results 6
Diffuse large B-cell lymphoma results 8
Follicular lymphoma results 11
Mantle cell lymphoma results 12
Survival by diagnosis 13
Summary of data completeness 14
Pathology Review Committee 14
Current projects 14
Completed projects 15
Data Access 15
Feedback and Enquiries 15
Glossary 15
Appendix 15

Introduction

This report contains a summary of data from the Lymphoma and Related Disease Registry (LaRDR) for the period 01 January 2016 to 05 November 2021. This includes information regarding T-cell lymphomas, diffuse large B-cell lymphoma (DLBCL), follicular lymphoma (FL), Hodgkin lymphoma (HL), mantle cell lymphoma (MCL) and other B-cell non-Hodgkin lymphomas. There are 4636 patients registered in the database. The total recruitment number includes patients diagnosed prior to 2016 for specific studies.

487 patients of the total number of patients were diagnosed with CLL. Data for chronic lymphocytic leukaemia have not been included and will be provided in a future report as these accrue in the new CLL data.

Only cases with a diagnosis within 6 months prior to HREC approval at the site are captured in this report, as per current HREC approval.

The usefulness of the registry relies on the quality and completeness of the data obtained from our partner sites. As cases and follow-up data continue to accrue, the registry will provide a better reflection of clinical practice in relation to the epidemiology, management and outcomes of lymphoma, both at your site and across Australia. A CLL-specific module, which collects the full clinical experience of CLL patients, has been integrated into LaRDR.

Completeness of different fields varies throughout the registry. We have included denominators for all fields to indicate the number of cases with populated data.

We thank you for your support of the registry.

Site status at time of report

Number of HREC approved sites: 30

Number of active sites: 23

Number of sites pending Ethics/Governance approval: 8

LaRDR sites across Australia and New Zealand

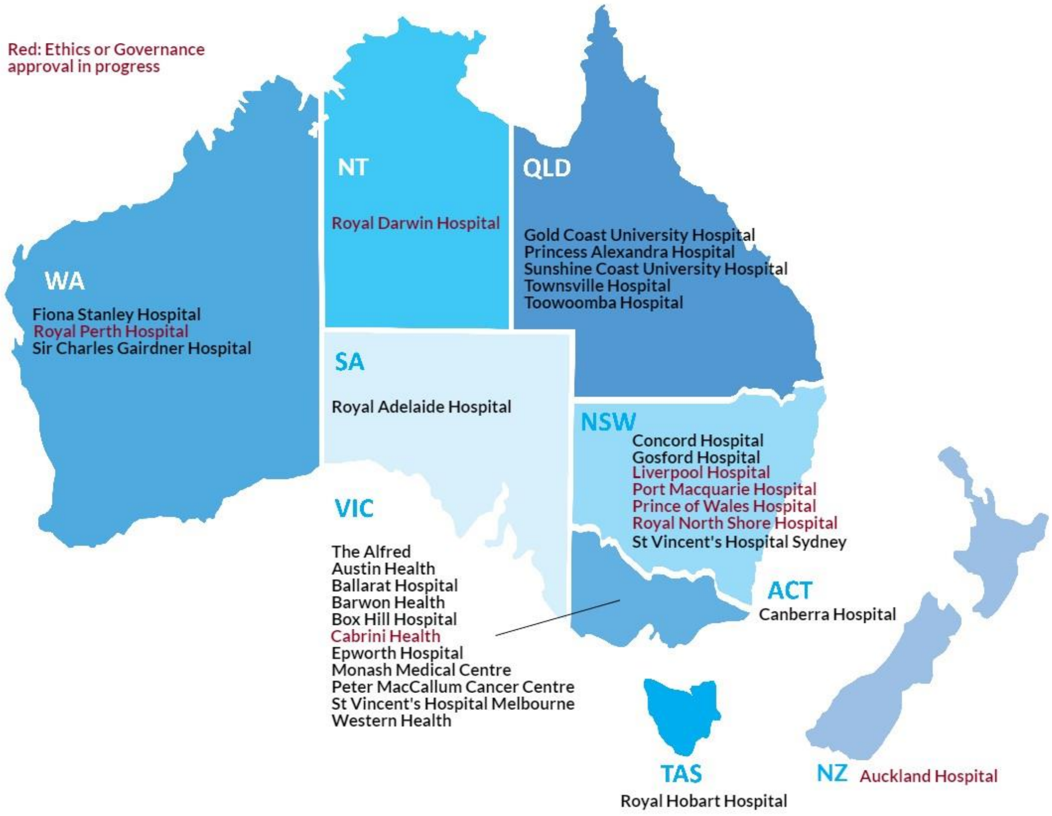

Figure 1. Participating sites in Australia and New Zealand. Active sites are shown in black text and sites that are pending Ethics or Governance approval are shown in red.

Case accrual

As of 05 November 2021, there were 4636 patients registered on the LaRDR.

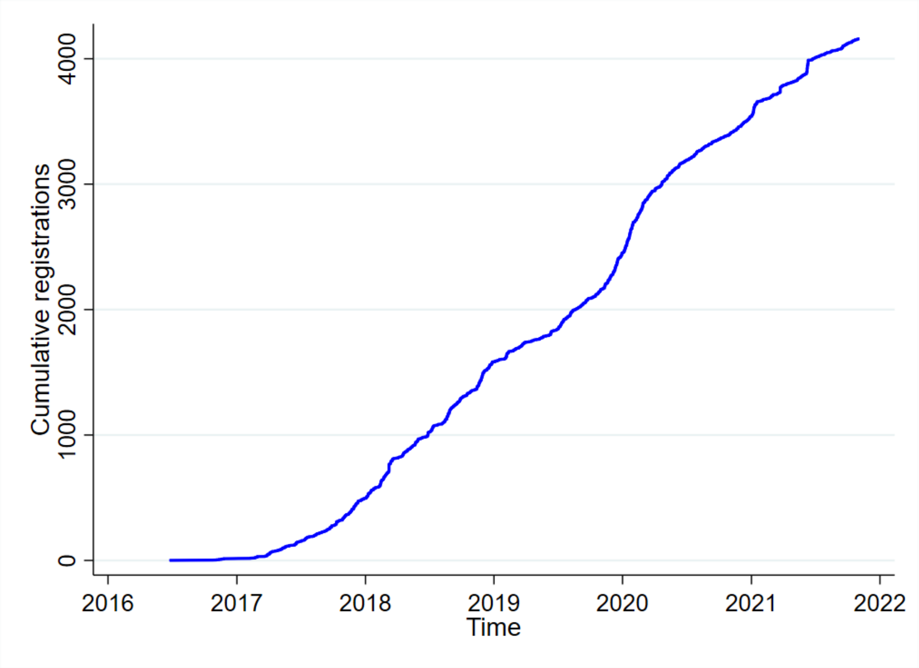

Figure 2. Cumulative patient registrations of all sites from 01 January 2016 to 05 November 2021.

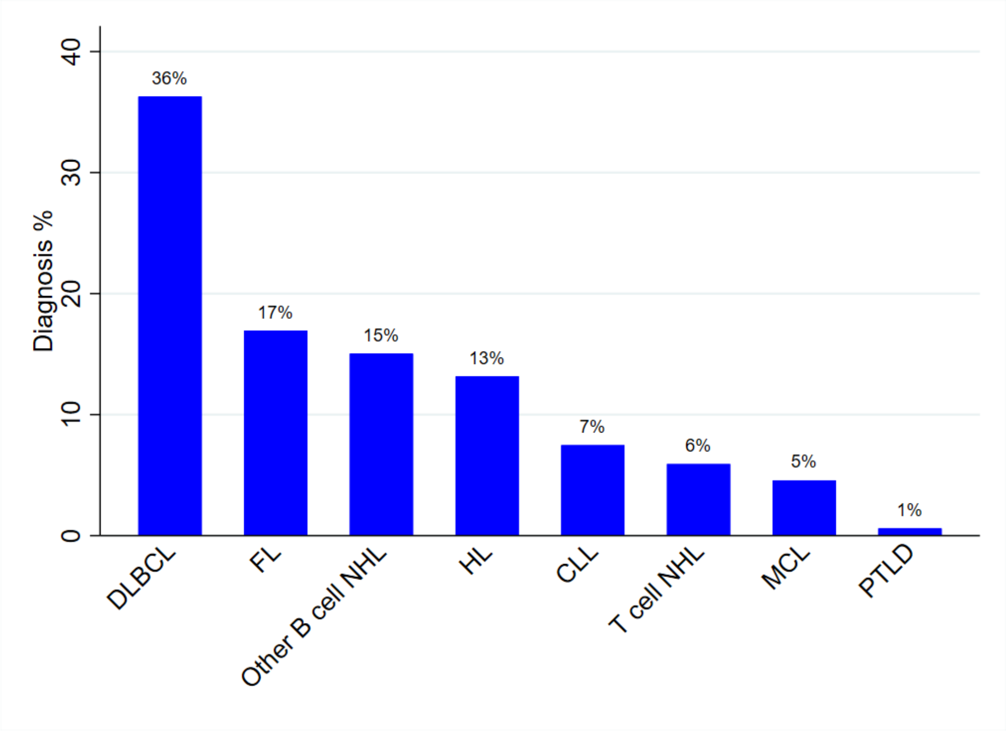

Figure 3. Breakdown of diagnoses.

Hodgkin lymphoma results

Table 1. Characteristics of patients diagnosed with Hodgkin lymphoma.

Factor	HL
Age at diagnosis (years), median (IQR)	36.6 (27.0, 56.1)
Age70	58/548 (10.6%)
Sex	
Male	313/548 (57.1%)
Female	235/548 (42.9%)
Staging (I-IV)	
I	47/515 (9.1%)
II	210/515 (40.8%)
III	96/515 (18.6%)
IV	162/515 (31.5%)
Staging (S)*	85/466 (18.2%)
Staging (X)*	96/465 (20.6%)
Staging (E)*	128/469 (27.3%)
Staging (A/B)	
A	314/503 (62.4%)
B	189/503 (37.6%)
ECOG performance	
0	358/473 (75.7%)
1	83/473 (17.5%)
2	20/473 (4.2%)
3	9/473 (1.9%)
4	3/473 (0.6%)
HLIPS	
0	24/501 (4.8%)
1	150/501 (29.9%)
2	131/501 (26.1%)
3	88/501 (17.6%)
4	67/501 (13.4%)
5	26/501 (5.2%)
6	13/501 (2.6%)
7	2/501 (0.4%)

* Refer to Glossary for explanations.

Table 2. Summary of treatment and response for patients with Hodgkin lymphoma.

Factor	HL
Watch and Wait	12/509 (2.4%)
Chemotherapy	436/509 (85.7%)
Clinical trial	38/509 (7.5%)
Radiotherapy	142/509 (27.9%)
Autologous stem cell transplant	5/509 (1.0%)
Allogeneic stem cell transplant	2/509 (0.4%)
Time to treatment (days), median (IQR)	21.0 (10.0, 35.0)
Response	
Complete response	335/412 (81.3%)

Partial response	33/412 (8.0%)
No response or stable disease	11/412 (2.7%)
Progressive disease	33/412 (8.0%)

Diffuse large B-cell lymphoma results

Table 3. Characteristics of patients diagnosed with diffuse large B-cell lymphoma.

Factor	DLBCL
Age at diagnosis (years), median (IQR)	68.2 (58.7, 75.9)
Age70	671/1510 (44.4%)
Sex	
Male	890/1510 (58.9%)
Female	620/1510 (41.1%)
Staging (I-IV)	
I	211/1222 (17.3%)
II	192/1222 (15.7%)
III	212/1222 (17.3%)
IV	607/1222 (49.7%)
Staging (S)*	160/1198 (13.4%)
Staging (X)*	255/1196 (21.3%)
Staging (E)*	665/1231 (54.0%)
Staging (A/B)	
A	884/1221 (72.4%)
B	337/1221 (27.6%)
ECOG performance	
0	610/1212 (50.3%)
1	399/1212 (32.9%)
2	129/1212 (10.6%)
3	52/1212 (4.3%)
4	22/1212 (1.8%)
Revised IPI score	
Very good	69/1003 (6.9%)
Good	416/1003 (41.5%)
Poor	518/1003 (51.6%)

Table 4. Summary of treatment and response of first line therapy for patients with diffuse large B-cell lymphoma.

Factor	DLBCL
Watch and Wait	9/1328 (0.7%)
Chemotherapy	1240/1328 (93.4%)
Clinical trial	54/1328 (4.1%)
Radiotherapy	264/1328 (19.9%)
Autologous stem cell transplant	50/1328 (3.8%)
Allogeneic stem cell transplant	4/1328 (0.3%)
Time to treatment (days), median (IQR)	14.0 (7.0, 26.0)
Response	
Complete response	787/1075 (73.2%)
Partial response	120/1075 (11.2%)
No response or stable disease	27/1075 (2.5%)
Progressive disease	141/1075 (13.1%)

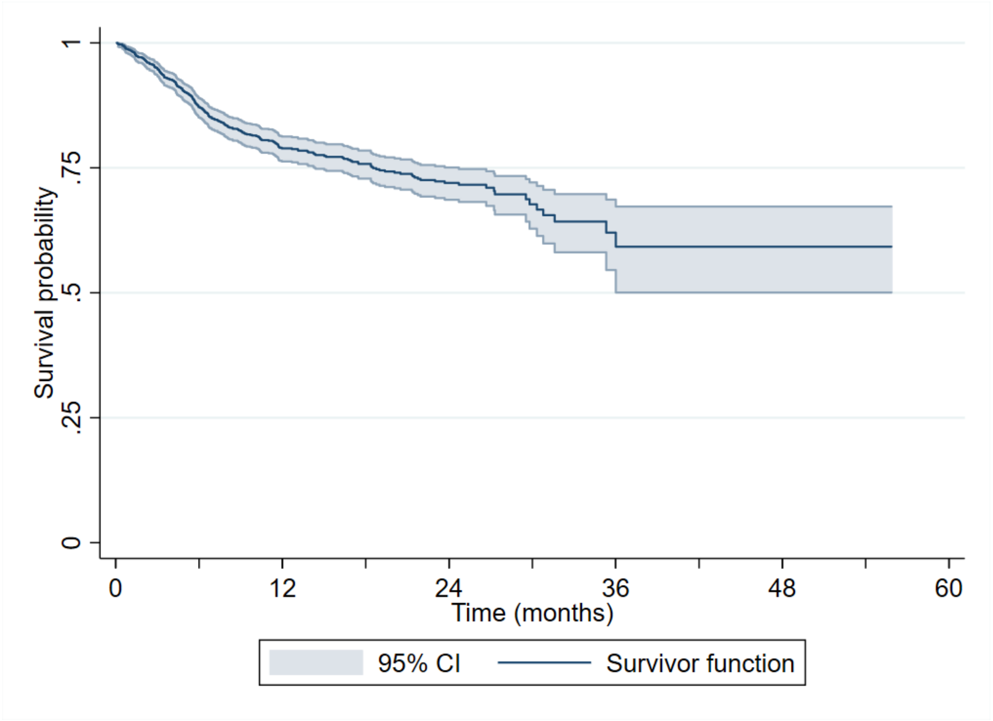

Figure 4. Progression-free survival of patients with DLBCL.

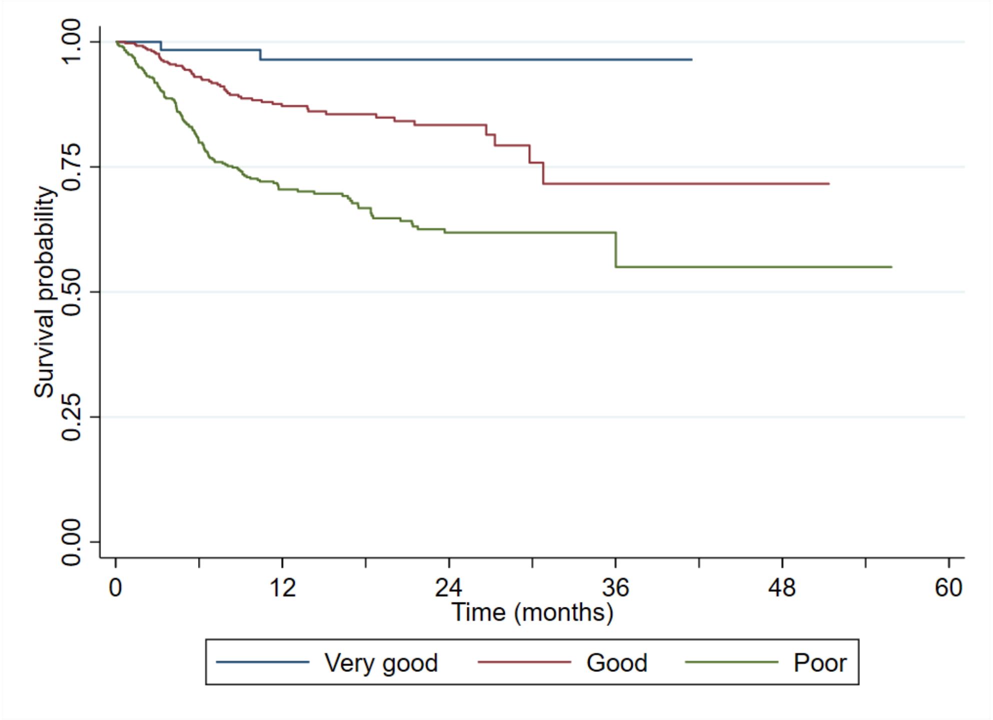

Figure 5. Progression-free survival of patients with DLBCL by R-IPI.

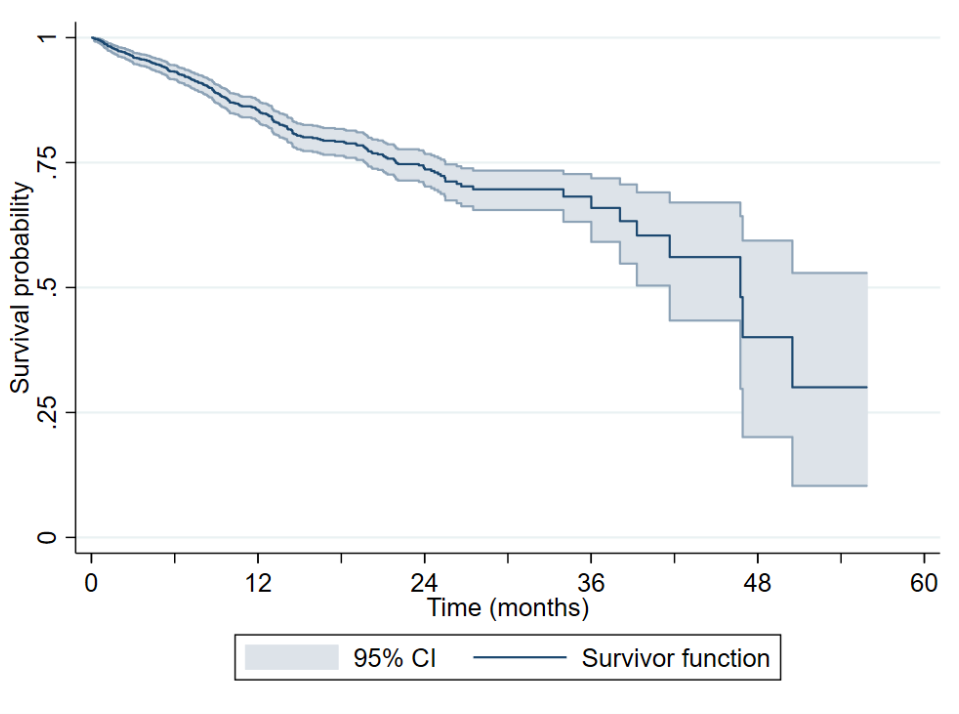

Figure 6. Overall survival of patients with DLBCL.

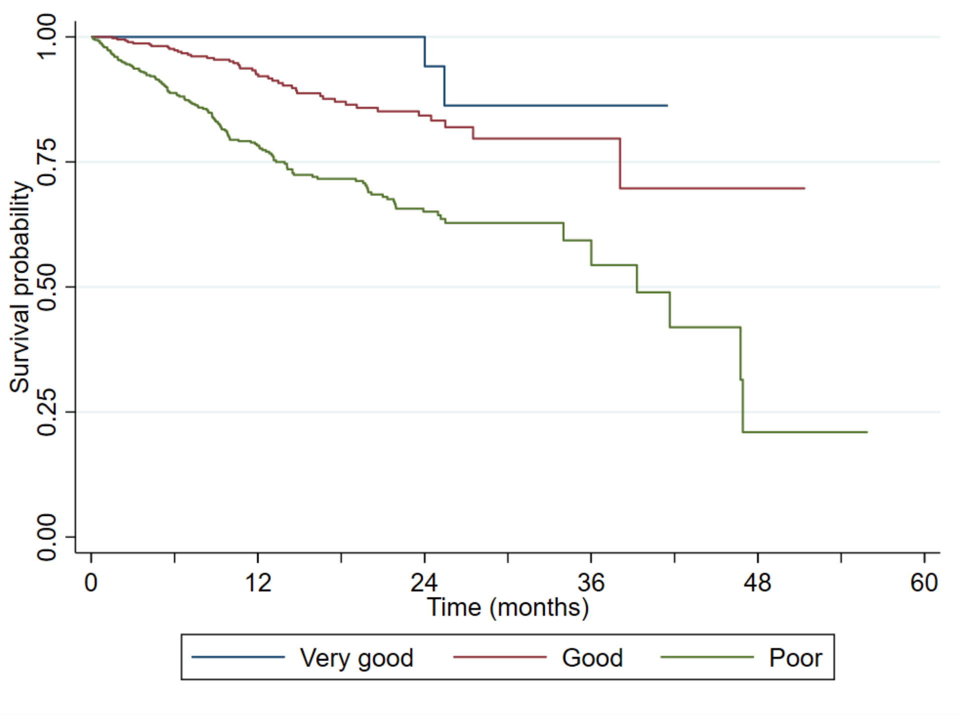

Figure 7. Overall survival of patients with DLBCL by R-IPI.

Follicular lymphoma results

Table 5. Characteristics of patients diagnosed with follicular lymphoma.

Factor	FL
Age at diagnosis (years), median (IQR)	65.3 (55.2, 73.6)
Age70	253/705 (35.9%)
Sex	
Male	384/704 (54.5%)
Female	320/704 (45.5%)
Staging (I-IV)	
I	121/645 (18.8%)
II	103/645 (16.0%)
III	199/645 (30.9%)
IV	222/645 (34.4%)
Staging (S)*	86/613 (14.0%)
Staging (X)*	109/616 (17.7%)
Staging (E)*	209/623 (33.5%)
Staging (A/B)	
A	525/644 (81.5%)
B	119/644 (18.5%)
ECOG performance	
0	406/615 (66.0%)
1	168/615 (27.3%)
2	33/615 (5.4%)
3	7/615 (1.1%)
4	1/615 (0.2%)
FLIPIRisk	
Low risk	229/576 (39.8%)
Intermediate risk	195/576 (33.9%)
High risk	152/576 (26.4%)

Table 6. Summary of treatment and response of first line therapy for patients with follicular lymphoma.

Factor	FL
Watch and Wait	168/585 (28.7%)
Chemotherapy	324/585 (55.4%)
Clinical trial	33/585 (5.6%)
Radiotherapy	123/585 (21.0%)
Autologous stem cell transplant	18/585 (3.1%)
Allogeneic stem cell transplant	0/585 (0.0%)
Time to treatment (days), median (IQR)	35.5 (18.0, 70.0)
Response	
Complete response	277/400 (69.3%)
Partial response	49/400 (12.3%)
No response or stable disease	52/400 (13.0%)
Progressive disease	22/400 (5.5%)

Mantle cell lymphoma results

Table 7. Characteristics of patients diagnosed with mantle cell lymphoma.

**Factor**	**MCL**
Age at diagnosis (years), median (IQR)	68.4 (60.2, 73.6)
Age70	81/190 (42.6%)
Sex	
Male	140/190 (73.7%)
Female	50/190 (26.3%)
Staging (I-IV)	
I	5/162 (3.1%)
II	15/162 (9.3%)
III	24/162 (14.8%)
IV	118/162 (72.8%)
Staging (S)*	63/164 (38.4%)
Staging (X)*	23/164 (14.0%)
Staging (E)*	81/165 (49.1%)
Staging (A/B)	
A	123/159 (77.4%)
B	36/159 (22.6%)
ECOG performance	
0	107/172 (62.2%)
1	46/172 (26.7%)
2	14/172 (8.1%)
3	4/172 (2.3%)
4	1/172 (0.6%)
MIPIRisk	
Low risk	47/157 (29.9%)
Intermediate risk	45/157 (28.7%)
High risk	65/157 (41.4%)

Table 8. Summary of treatment and response of first line therapy for patients with mantle cell lymphoma.

**Factor**	**MCL**
Watch and Wait	14/158 (8.9%)
Chemotherapy	128/158 (81.0%)
Clinical trial	15/158 (9.5%)
Radiotherapy	7/158 (4.4%)
Autologous stem cell transplant	45/158 (28.5%)
Allogeneic stem cell transplant	4/158 (2.5%)
Time to treatment (days), median (IQR)	21.0 (11.0, 45.0)
Response	
Complete response	76/115 (66.1%)
Partial response	17/115 (14.8%)
No response or stable disease	9/115 (7.8%)
Progressive disease	13/115 (11.3%)

Survival by diagnosis

The graphs below show the progression-free and overall survival of patients who have the information entered into the database.

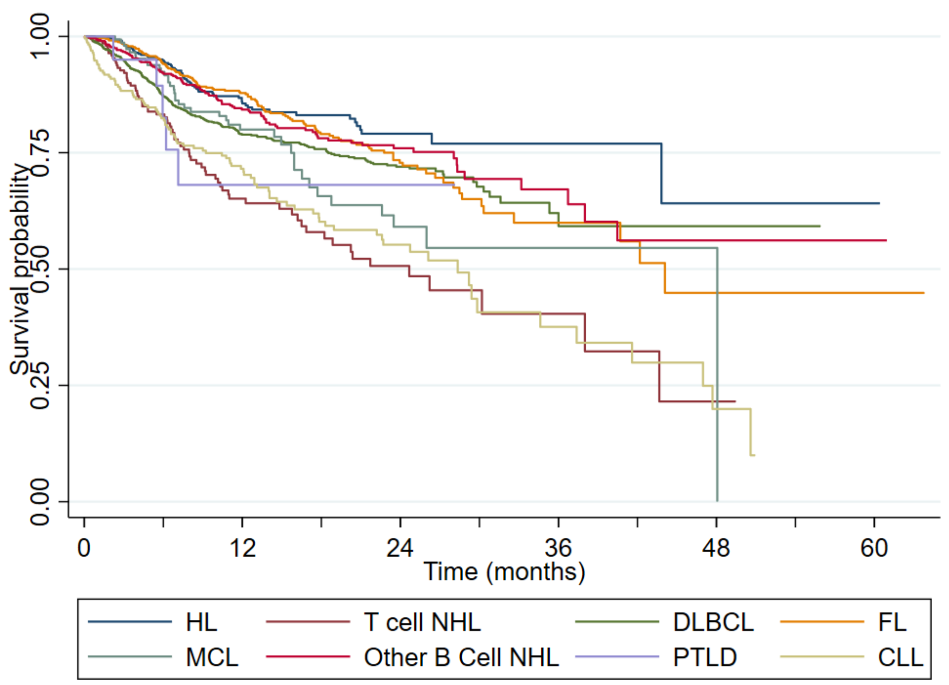

Figure 8. Progression-free survival of patients by diagnosis.

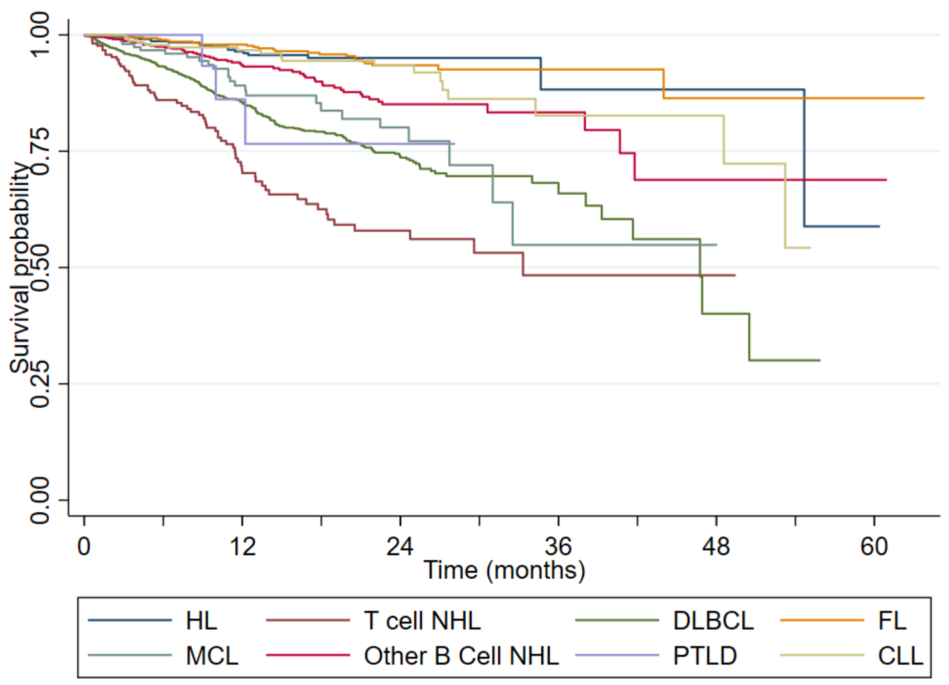

Figure 9. Overall survival of patients by diagnosis.

Summary of data completeness

Table 9. Summary of data completeness for treatment and review forms.

Factor	All sites
Treatment delivered	3246/4161 (78.0%)
Treatment response	2798/3246 (86.2%)
6 month review	2968/4075 (72.8%)
12 month review	2264/3856 (58.7%)
24 month review	1272/3181 (40.0%)

*The total number of patients recruited may not have had their reviews conducted.

Pathology Review Committee

The Pathology Review Committee is chaired by A/Prof Dipti Talaulikar. The group oversees diagnostic aspects of registry cases.

Current projects

Various research projects have already been established using the data from the LaRDR registry. These include the:

- A review of the patterns of treatment and survival outcomes for patients with large B-cell lymphoma in Australia
- A review of the patterns of treatment and survival outcomes for patients with mantle cell lymphoma in Australia
- Characteristics of Hodgkin Lymphoma in Australia
- Epidemiology and Management of Chronic Lymphocytic Leukemia Patients in Australia
- Factors influencing outcome in Richter's Transformation; results of a multi-centre retrospective study on Richter's Transformation of Chronic Lymphocytic Leukemia or Small Lymphocytic Lymphoma to Diffuse Large B-cell Lymphoma or Hodgkin Lymphoma
- Front-line Treatment of Elderly Patients with Classical Hodgkin Lymphoma: A Multicentre Retrospective Analysis from the ALA
- GLOW Study - Outcomes for Stage I-IV Nodular Lymphocyte Predominant Hodgkin Lymphoma: A Multi-Institutional Retrospective Review
- HoLISTIC Consortium - Hodgkin Lymphoma International Study for Individual Care
- Movember cancer registries platform
- Real-world Experience of Polatuzumab Vedotin in Relapsed or Refractory Diffuse Large B Cell Lymphoma
- T-Cell 2.0 – observational international registry of patients with newly diagnosed peripheral T-cell lymphoma.

Some of these projects offer project-specific funding.

- Ibrutinib follow-up named patient program (NPP) analyses for CLL and MCL (now recruiting)

- Immunoglobulin use and outcomes in Chronic lymphocytic leukaemia And Non-Hodgkin lymphoma – The ICAN Study (now recruiting)

Completed projects

- GELF criteria project
- Melbourne Genomics Health Alliance Lymphoma Flagship
- WhiMSICAL – Waldenström Macroglobulinaemia Study Involving Cart-wheeL

Data Access

If you have a registry-based project idea, and/or would like to access data from the registry, please contact us using the email address listed at the end of this document.

Feedback and Enquiries

The usefulness of these reports will be enhanced by the quality and completeness of the data. Thank you for your contribution to the LaRDR and your ongoing support of the registry. We welcome your feedback and suggestions to enhance the utility of these reports

Email: sphpm-lymphoma@monash.edu.

Website: www.lardr.org

Glossary

- Staging (S) - Spleen involvement
- Staging (X) - Bulky disease
- Staging (E) - Extranodal involvement

Appendix

This appendix shows the groupings of classifications that are used for the diagnoses. The diagnoses that are not included here do not have further classifications.

**Diagnosis**	**ICD-O-3 categories**
Hodgkin's Lymphoma	• 9663/3 Nodular sclerosis classical Hodgkin lymphoma<br>• 9650/3 Classical Hodgkin lymphoma<br>• 9652/3 Mixed cellularity classical Hodgkin lymphoma<br>• 9659/3 Nodular lymphocyte predominant Hodgkin lymphoma<br>• 9651/3 Lymphocyte-rich classical Hodgkin lymphoma<br>• 9653/3 Lymphocyte-depleted classical Hodgkin lymphoma
T cell NHL	• 9719/3 Extranodal NK/T cell lymphoma, nasal type<br>• 9714/3 Anaplastic large cell lymphoma, ALK positive

	• 9705/3 Angioimmunoblastic T-cell lymphoma<br>• 9702/3 Peripheral T-cell lymphoma, NOS<br>• 9827/3 Adult T-cell leukaemia/lymphoma<br>• 9717/3 Enteropathy-associated T-cell lymphoma<br>• 9716/3 Hepatosplenic T-cell lymphoma<br>• 9708/3 Subcutaneous panniculitis-like T-cell lymphoma<br>• 9702/3 Anaplastic large cell lymphoma, ALK negative
DLBCL	• 9680/3 Diffuse large B-cell lymphoma (DLBCL), NOS<br>• 9680/3 Primary DLBCL of the CNS<br>• 9680/3 EBV positive DLBCL of the elderly<br>• 9680/3 Primary cutaneous DLBCL, leg type<br>• 9680/3 DLBCL associated with chronic inflammation
Follicular lymphoma	• 9690/3 Follicular lymphoma
MCL	• 9673/3 Mantle cell lymphoma
Other B cell NHL	• 9680/3 B-cell lymphoma, unclassifiable, with features intermediate between diffuse large B-cell lymphoma and Burkitt lymphoma<br>• 9687/3 Burkitt lymphoma<br>• 9699/3 Nodal marginal zone lymphoma<br>• 9699/3 Extranodal marginal zone lymphoma of mucosa-associated lymphoid tissue (MALT lymphoma)<br>• 9596/3 B-cell lymphoma, unclassifiable, with features intermediate between diffuse large B-cell lymphoma and classical Hodgkin lymphoma<br>• 9679/3 Primary mediastinal (thymic) large B-cell lymphoma<br>• 9761/3 Waldenstrom macroglobulinemia<br>• 9689/3 Splenic marginal zone lymphoma<br>• 9688/3 T-cell/histiocyte rich large B-cell lymphoma<br>• 9712/3 Intravascular large B-cell lymphoma<br>• 9671/3 Lymphoplasmacytic lymphoma<br>• 9735/3 Plasmablastic lymphoma<br>• 9597/3 Primary cutaneous follicle centre lymphoma<br>• 9678/3 Primary effusion lymphoma<br>• 9766/1 Lymphomatoid granulomatosis<br>• 9737/3 ALK positive large B-cell lymphoma
PTLD	• 9971/3 Polymorphic post-transplant lymphoproliferative disorder
CLL	• 9823/3 Chronic lymphocytic leukaemia/small lymphocytic lymphoma
